# Supplementary figures and images for: Network Analysis Reveals Distinct Clinical Syndromes Underlying Acute Mountain Sickness
Source: PLoS One. 2014 Jan 22;9(1):e81229. doi: 10.1371/journal.pone.0081229 (PMC3898916; doi:10.1371/journal.pone.0081229)

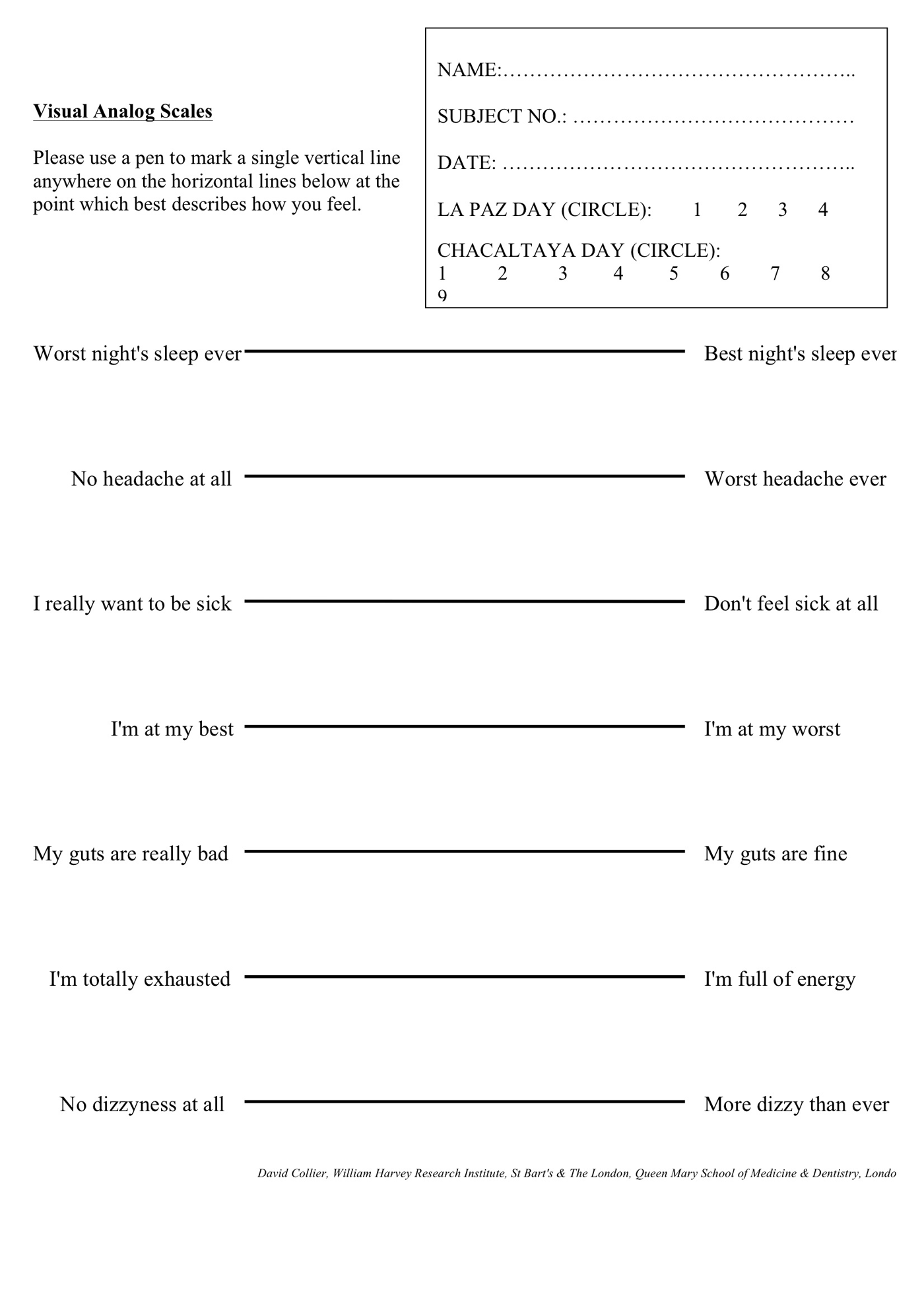

Supplement: Figure S1 — VAS questionnaire form. Questionnaire given to subjects to record VAS scores relating to symptoms experienced at altitude on the Apex 2 expedition. The same form was used by participants on the Kilimanjaro expedition. (TIF) [file pone.0081229.s001.tif]

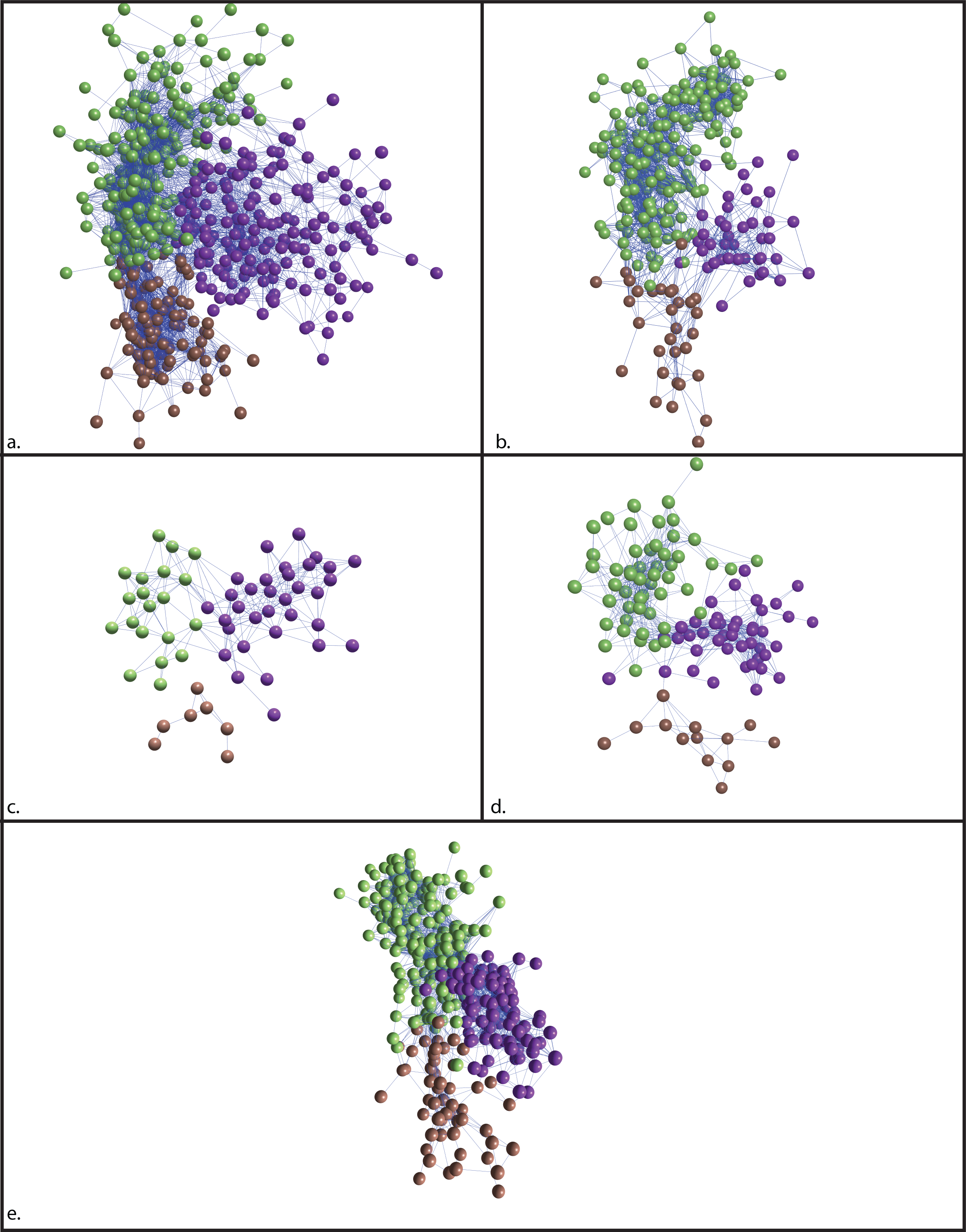

Supplement: Figure S2 — Supplementary symptom networks. The network graph created in Biolayout 3D Express, which incorporated data from all 1045 questionnaires and is displayed as Figure 1 , was reproduced using differing questionnaire inclusion criteria. These all produced at least two distinct clusters when clustered using a MCL inflation value of 1.4. (A) includes questionnaires from Apex 2 Expedition subjects only (n = 869); (B) includes only questionnaires from subjects not taking either sildenafil or antioxidant supplementation (n = 523); (C) includes questionnaires from Kilimanjaro subjects only (n = 176); (D) includes only questionnaires from subjects at a single time point (day 3 of the Apex 2 expedition, and all Kilimanjaro participants, n = 269). (E) includes Apex 2 questionnaires, in which the sleep score from the following night was used in place of that from the preceding night (n = 625). (TIFF) [file pone.0081229.s002.tiff]

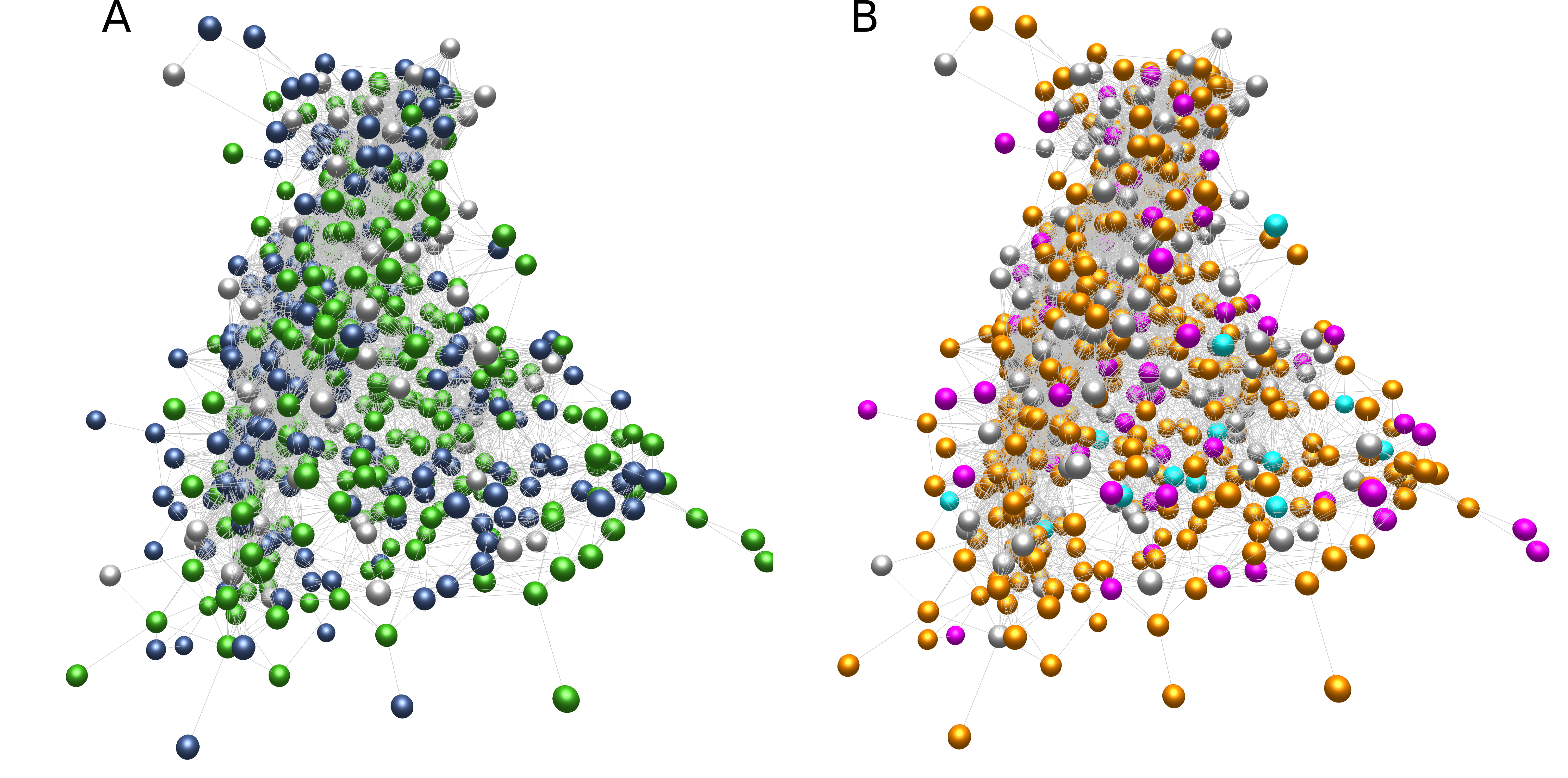

Supplement: Figure S3 — Age and sex distribution in symptom network. Each node (coloured sphere) represents a VAS questionnaire, connected by weighted lines, which represent correlations between similar symptom profiles. Nodes are connected with each other if the Pearson correlation coefficient between them exceeds 0.95. (A) Sex distribution of nodes, with questionnaires completed by males denoted by green nodes, those by females by blue, and missing demographic data by grey nodes; (B) Age distributions of nodes, with questionnaires completed by under 21 year olds represented by orange nodes, 22–25 year olds by pink nodes, and those completed by over 26 years old by cyan. Missing data are represented by grey nodes. (TIFF) [file pone.0081229.s003.tiff]
